# Effects of 405-nm LED Treatment on the Resistance of *Listeria monocytogenes* to Subsequent Environmental Stresses

**DOI:** 10.3389/fmicb.2019.01907

**Published:** 2019-08-16

**Authors:** Shenmin Kang, Yujie Meng, Xiaomeng Cheng, Junhong Tu, Du Guo, Yunfeng Xu, Sen Liang, Xiaodong Xia, Chao Shi

**Affiliations:** ^1^College of Food Science and Engineering, Northwest A&F University, Yangling, China; ^2^College of Food and Bioengineering, Henan University of Science and Technology, Luoyang, China; ^3^Beijing Advanced Innovation Center for Food Nutrition and Human Health, Beijing Technology and Business University, Beijing, China

**Keywords:** *L. monocytogenes*, LED, environmental stress resistance, cell membrane integrity, resistance gene

## Abstract

*Listeria monocytogenes* can persist under a wide range of stress conditions, contributing to its ubiquitous distribution and unique pathogenic traits. Light from light-emitting diodes (LEDs) has recently been shown to inactivate various pathogens. Thus, the aim of the present study was to evaluate the effects of light treatment using a 405-nm LED on the subsequent resistance of *L. monocytogenes* to environmental stresses, including oxidative stress, ultraviolet (UV) irradiation, low temperature, osmotic pressure, simulated gastric fluid (SGF), and bile salts. Following 405-nm LED illumination at 4°C for 150 min, the survival of *L. monocytogenes* was examined after exposure to oxidative stress (0.04% H_2_O_2_), UV irradiation (253.7 nm), low temperature (4°C), osmotic pressure (10, 15, or 20% NaCl), SGF (pH 2.5), or bile salts (2%). The mechanisms responsible for changes in stress tolerance were identified by assessing the transcriptional responses and membrane integrity of *L. monocytogenes*. The 405-nm LED treatment reduced the resistance of *L. monocytogenes* to all the stresses tested. Reverse transcription quantitative real-time polymerase chain reaction analysis indicated that the transcription of multiple genes associated with stress resistance, including *betL*, *gbuA*, *oppA*, *fri*, *bsh*, and *arcA*, was reduced by 405-nm LED. Confocal laser scanning microscopy revealed that 405-nm LED treatment disrupted the integrity of the *L. monocytogenes* cell membrane compared with untreated bacteria. Therefore, 405-nm LED illumination appears to reduce the resistance of *L. monocytogenes* to various stress conditions. These findings suggest that 405-nm LED treatment could be used to effectively prevent and/or control with *L. monocytogenes* contamination along the entire food-processing chain, from production to consumption.

## Introduction

*Listeria monocytogenes* is a foodborne, facultative, intracellular human pathogen that is highly resistant to various stress conditions (Bucur et al., 2018). It crosses both the blood-brain and intestinal barriers, causing life-threatening infections. *L. monocytogenes* is the most common cause of the serious foodborne disease listeriosis, a sporadic bacterial infection that affects many animal species, including humans (Scallan et al., 2011). Older adults, pregnant women, newborns, and immunodeficient individuals are especially susceptible to listeriosis (Lecuit, 2007). Patients infected with *Listeria* often suffer meningitis, sepsis, and other central nervous system infections, while listeriosis can also cause spontaneous abortion in pregnant women (Rocourt, 2019). In 2016, 270 deaths attributable to listeriosis were reported across 19 member states of the European Union (EU). In the same year, the overall EU notification rate for listeriosis was 0.46 cases per 100,000 population, with a case-fatality rate of 17.7% (Boelaert et al., 2016). Many studies have shown that rivers, sewage, and natural soils are reservoirs of *L. monocytogenes* (Schaffter and Parriaux, 2002; Watkins and Sleath, 2010). However, processed foods are common sources of infection, including raw milk products, cold meats, pâtés, and ready-to-eat chilled products (Lecuit, 2007; Allerberger and Wagner, 2010), although unprocessed foods, such as fruit and vegetables, have also been identified as vectors (Garner and Kathariou, 2016).

*Listeria monocytogenes* can tolerate oxidative environments and was reported to survive for more than 15 min after exposure to the oxidative agent cumene hydroperoxide (13.8 mM) (Ferreira et al., 2001). Studies have shown that the persistence of *L. monocytogenes* under oxidative conditions is related to stress regulators encoded by *sigB* and *perR*, antioxidant genes *sod*, *kat*, and *fri*, and the DNA repair gene *recA* (Fisher et al., 2000; Bredèche et al., 2001; Rea et al., 2005; Fiorini et al., 2008; Van der Veen and Abee, 2010). Refrigeration is commonly used to extend the shelf-life of food products. Various studies have demonstrated that *L. monocytogenes* can survive and grow at refrigeration temperatures (2–4°C); although, at these temperatures, its doubling time can be 50 h or more (Feng et al., 2010). Bayles et al. (1996) showed that *L. monocytogenes* contains 12 cold-shock proteins, that allow it to survive and grow at low temperatures. *L. monocytogenes* strains can also adapt to highly osmotic environments, surviving for 150 days in pure salt at ambient temperature (Besse et al., 2010) and thriving at NaCl concentrations of up to 14% (w/v) (Farber et al., 2010). The mechanism underlying the response of *L. monocytogenes* to high osmotic pressure depends on the synthesis or uptake of compatible solutes, including glycine betaine, proline, and carnitine, to balance its internal and external environments (Gardan et al., 2003; Sleator et al., 2003). *L. monocytogenes* is also resistant to ultraviolet (UV) irradiation, acid, and bile salts (Gabriel and Nakano, 2009; Feng et al., 2010; Chorianopoulos et al., 2011). The multifaceted environmental tolerance of *L. monocytogenes* increases its ability to survive the food processing chain, increasing the risk of human infection and the difficulty of eliminating it.

Light emitting diodes (LEDs) are semiconductor devices that emit visible light. The many advantages of LEDs include low energy consumption, longevity, and non-polluting action (Murdoch et al., 2010). In recent years, researchers have investigated the potential utility of specific-wavelength LEDs to eliminate foodborne pathogens. Studies have shown that illumination with a 405-nm LED has strong bacteriostatic effects on Gram-positive bacteria, including *Bacillus cereus* and *Staphylococcus aureus* (Kim et al., 2015), and an antibacterial effect on *Escherichia coli* O157:H7 on the surface of fresh-cut mango (Kim et al., 2016). Endarko et al. (2012) showed that 405-nm LED treatment exerted a strong germicidal effect on *L. monocytogenes*. Several studies have proposed different mechanisms for the killing effects of LED treatment on bacterial cells. One group suggested that after bacteria are exposed to blue-violet light in the presence of oxygen, an endogenous porphyrin compound can absorb light and then excite electrons, producing reactive oxygen species (ROS) (Dai et al., 2012). ROS such as superoxide ions may cause cytotoxicity by reacting with intracellular components including DNA and proteins, resulting in bacterial death (Luksiene, 2003). Other studies have shown that the antibacterial effect of 405-nm LED treatment may be partly attributed to the physical damage caused to the bacterial cell membrane (Kim et al., 2015). However, few studies have examined the effects of 405-nm LED treatment on the resistance of *L. monocytogenes* to environmental stresses, or what the underlying mechanisms may be.

The aim of the current study was to investigate the effects of 405-nm LED treatment on the resistance of *L. monocytogenes* to environmental stresses. Differences in the resistance of illuminated versus unilluminated bacteria to oxidative stress, UV irradiation, osmotic pressure, low temperature, simulated gastric fluid (SGF), and bile salts were detected. The effects of LED illumination on the integrity of the *L. monocytogenes* cell membrane was also examined using confocal laser scanning microscopy (CLSM). Finally, reverse transcription quantitative real-time polymerase chain reaction (RT-qPCR) was used to investigate the effects of 405-nm LED treatment on the transcription of resistance-related genes in *L. monocytogenes*.

## Materials and Methods

### Bacterial Strains and Culture Conditions

*Listeria monocytogenes* strain ATCC 19115 was purchased from the American Type Culture Collection (Manassas, VA, United States) and it was used for further experiments because it is commonly used in studies about environmental resistance of *L. monocytogenes* and it contains phenotypic and genotypic characteristics tested in the following experiments. The strain was stored at -80°C. Bacteria were subcultured on trypticase soy agar (TSA; Land Bridge, Beijing, China) for 30 h at 37°C. A single colony was then inoculated into 30 mL of sterile tryptic soy broth (TSB; Land Bridge) and incubated with shaking at 130 rpm for 18–24 h at 37°C. This working culture was then centrifuged at 8000 × *g* for 10 min at 4°C and the cell pellet was washed twice with phosphate-buffered saline (PBS; pH 7.4). The pellet was then resuspended in PBS to produce a concentrated cell culture (approximately 10^9^ colony-forming units (CFU)/mL).

### LED Illumination

The 405-nm LED was purchased from Shenzhen Boya Technology Co., Ltd. (Shenzhen, China). The LED illumination system was designed as reported by Ghate et al. (2013). Briefly, the LED was surrounded by acrylonitrile butadiene styrene housing and a cooling fan and a heat sink were attached to cool the apparatus during the illumination process. To protect the LED from excessive current, 5 Ω of resistance was linked into the circuit. The distance between the LED source and the bacterial suspension, contained in a sterile Petri dish (90 × 15 mm), was 4.5 cm to ensure that no visible color fading was generated on the suspension during LED illumination. A digital thermometer (DTM-280; Shanghai Shuangqiao Instrument Co., Ltd., Shanghai, China) was used to monitor fluctuations in the temperature of the bacterial suspension during LED illumination over 4 h. To compensate for the temperature increase during LED illumination, the unilluminated *L. monocytogenes* cultures were placed in an incubator at 5.4°C in the dark, compensating for the increase in temperature measured in the illuminated culture (measured as 1.4°C). The dose of LED illumination received by each bacterial sample was calculated using the formula: E = Pt, where E is dose (energy density) in J/cm^2^, P is the irradiance (power density) in mW/cm^2^, and t is the time in seconds (Maclean et al., 2009).

### Bacterial Inoculation and LED Illumination

Aliquots (2 mL) of bacterial suspension (prepared as described in section “Bacterial Strains and Culture Conditions”) were diluted in 18 mL of PBS to produce the test cultures. Test cultures were aspirated into sterile Petri dishes (90 × 15 mm) and illuminated using 405-nm LED (18.9 ± 1.4 mW/cm^2^) for 240 min at 4°C. Unilluminated samples were placed in an incubator at 5.4°C. At time points 0, 30, 90, 150, 180, and 240 min, aliquots of both illuminated and unilluminated samples were collected diluted in sterile PBS, and plated onto TSA plates. The plates were incubated for 24–48 h at 37°C for colony enumeration.

### Determination of Resistance to Environmental Stress Conditions

*Listeria monocytogenes* ATCC 19115 treated with 405-nm LED for 150 min at 4°C was used to study resistance to environmental stresses. Unilluminated samples were incubated for 150 min at 5.4°C. Following all treatments, samples were serially diluted in PBS and then plated onto appropriate agar plates for colony enumeration. Final bacterial counts were calculated as log CFU/mL.

#### Resistance of *L. monocytogenes* to Oxidative Stress

To evaluate the survival of *L. monocytogenes* in the presence of 0.04% (v/v) H_2_O_2_, unilluminated and LED-illuminated samples were transferred to separate sterile tubes. An equal volume of PBS containing 0.08% (v/v) H_2_O_2_ was added to each tube and gently mixed before the cultures were incubated at 25°C. Samples were collected from each tube at 5 min intervals for 20 min and plated on TSA.

#### UV Resistance of *L. monocytogene*s

To examine the survival of *L. monocytogenes* following exposure to UV irradiation, unilluminated and LED-illuminated samples were aspirated into sterile Petri dishes (90 × 15 mm) and exposed to UV irradiation. An 8-W UV light (UV-C; 253.7 nm) was purchased from Haining Haishi Lighting Co., Ltd. (Haining, China). The distance between the UV source and the bacterial suspensions was 55 cm. After UV irradiation for 0, 20, 40, 60, 120, 180, or 240 s, cultures were serially diluted and plated on TSA.

#### Cold Resistance of *L. monocytogene*s

The cold resistance of *L. monocytogenes* was determined by incubating unilluminated and LED-illuminated *L. monocytogenes* at 4°C. Cultures were sampled at 0, 4, 24, 48, and 96 h post-cold-exposure and plated on TSA.

#### Resistance of *L. monocytogenes* to Osmotic Pressure

To evaluate the effects of osmotic pressure on *L. monocytogenes* survival, NaCl was added to the unilluminated and LED-illuminated samples to a final concentration of 10, 15, or 20% (w/v). Cultures were then incubated at 25°C and samples were collected at 0, 20, 30, 40, 60, or 120 min and plated on TSA for colony enumeration.

#### SGF Resistance of *L. monocytogene*s

Simulated gastric fluid (pH 2.5) was prepared according to Yang et al. (2015) and consisted of 8.3 g proteose peptone (Solarbio, Beijing, China), 0.6 g KH_2_PO_4_, 2.05 g NaCl, 0.37 g KCl, 0.11 g CaCl_2_, 0.05 g oxgall (Sigma Aldrich, St. Louis, MO, United States), 1 g lysozyme (Solarbio), and 13.3 mg pepsin (1:3000; Solarbio). All of the compounds were dissolved in deionized water and autoclaved together, except for the oxgall, lysozyme, and pepsin, which were sterilized by filtration (0.25 μm pore size). The solution was adjusted to pH 2.5 with 5.0 N HCl.

The acid resistance of the *L. monocytogenes* strains was determined according to Yang et al. (2015). SGF was inoculated with unilluminated or LED-illuminated samples at a ratio of 9:1 and incubated with shaking (130 rpm) at 37°C. Aliquots were collected from each sample at 0, 2, 5, 10, 20, or 30 min post-inoculation and plated on TSA.

#### Resistance of *L. monocytogenes* to Bile Salts

To evaluate the survival of *L. monocytogenes* in the presence of 2% (w/v) bile salts, bile salt solution (Sigma Aldrich) was added to the unilluminated and LED-illuminated samples to a final concentration of 2% (w/v), placed on a shaker (130 rpm), and incubated at 37°C. At 0, 30, 60, 120, 180, or 240 min, the cultures were serially diluted and plated on TSA. Plates were incubated at 37°C for 24–48 h for colony enumeration.

### Effect of 405-nm LED on *L. monocytogenes* Treatment Cell Membrane Integrity

*Listeria monocytogenes* ATCC 19115 was illuminated with 405-nm LED at 4°C for 90 or 150 min. Unilluminated *L. monocytogenes* was incubated at 5.4°C for 90 or 150 min. Both unilluminated and LED-illuminated samples were then centrifuged at 10,000 × *g* for 2 min at 4°C to obtain the cell pellets, which were resuspended in 0.85% (w/v) NaCl. The reagents SYTO 9 and propidium iodide (PI) were prepared according to the manufacturer’s instructions and mixed in equal proportions. A 3 μL aliquot of this mixture was then added to a 1-mL aliquot of each cell suspension in 0.85% (w/v) NaCl and the cells were incubated for 10 min in the dark. The stained cells were examined by CLSM (A1 confocal laser microscope; Nikon, Tokyo, Japan) under 400× magnification.

### Effect of 405-nm LED Treatment on Gene Transcription in *L. monocytogene*s

*Listeria monocytogenes* ATCC 19115 was illuminated using the 405-nm LED at 4°C for 150 min. Unilluminated *L. monocytogenes* was incubated at 5.4°C for 150 min. Cultures were then centrifuged (5000 × *g*, 5 min, 4°C) and resuspended in PBS. Total RNA was extracted using a RNAprep Pure Kit (Tiangen, Beijing, China) according to the manufacturer’s instructions. RNA concentrations were measured using a nucleic acid and protein spectrophotometer (Nano-200; Aosheng Instrument Co., Ltd., Hangzhou, China). The isolated RNA samples were then reverse transcribed into cDNA, and RT-qPCR was performed as per the method of Shi et al. (2018). All samples were analyzed in triplicate, and gene expression was normalized against that of the endogenous control (16S rRNA gene). The primers used in this study are listed in Table 1. The samples were analyzed using the IQ5 system (Bio-Rad Laboratories, Hercules, CA, United States) and the expression of the target genes and the 16S rRNA gene was determined as described above. The relative expression levels of the target genes was determined using the 2^–ΔΔ*Ct*^ method.

**TABLE 1:** Primers used in this study for RT-qPCR analyses.

**Gene**	**Sequence of primers (5′–3′)**
16S rRNA	F: GATGCATAGCCGACCTGAGA R: TGCTCCGTCAGACTTTCGTC
*sigB*	F: CATTGCTGATTTCATCGGTGT R: CCACCAACAACATCTAATAAGG
*betL*	F: TGGGCTTGGTGGCTTTCT R: TCGCTAAACTTGAAAGACCAG
*gbuA*	F: GTCCATGCAGCGGAAGTTTC R: CATCTTCGGTTACAGCAATCG
*arcA*	F: TCTTGTCGGTGTATCTGAGCG R: CCATGGTGAAAACCGTATCC
*bsh*	F: TATGATAATCCTGTTGGCGTGTT R: GAAGATAAATCGCCAGGTAAGC
*oppA*	F: CGGTGGAGATATCGTAGCTG R: AACTCGAAAGGTCCATTGAA
*fri*	F: CACCAAGTAGCGAACCTAAACG R: TAAGCGTTCTGCTACTTCGTCC

### Statistical Analysis

All experiments were performed in triplicate. The data are expressed as means ± standard deviations and were statistically analyzed using SPSS version 19.0 (SPSS Inc., Chicago, IL, United States). Significant differences were determined using the LSD method. The differences between groups were considered significant at *P* < 0.05 and extremely significant at *P* < 0.01.

## Results

### Temperature of *L. monocytogenes* Suspension During LED Illumination

The temperature of the *L. monocytogenes* suspension was measured every 1 min for 240 min during LED illumination. After 70 min of measurement, regular fluctuations in temperature were recorded. Overall, the temperature rose by an average of 1.4°C when the *L. monocytogenes* suspension at the set temperature of 4°C was illuminated using the 405-nm LED. The regular fluctuations continued until the end of measurement period (Supplementary Figure S1). Therefore, when the test cells were illuminated for 150 min (total dose of 170 J/cm^2^) at 4°C, the unilluminated *L. monocytogenes* cells were placed in an incubator at 5.4°C to compensate for the increase in temperature in the illuminated suspension.

### Survival of *L. monocytogenes* Following 405-nm LED Treatment

Following illumination using the 405-nm LED (18.9 ± 1.4 mW/cm^2^) for 90, 150, or 180 min, the total doses of LED illumination received by the bacterial suspensions were 102, 170, and 204 J/cm^2^, respectively.

**FIGURE 1:**
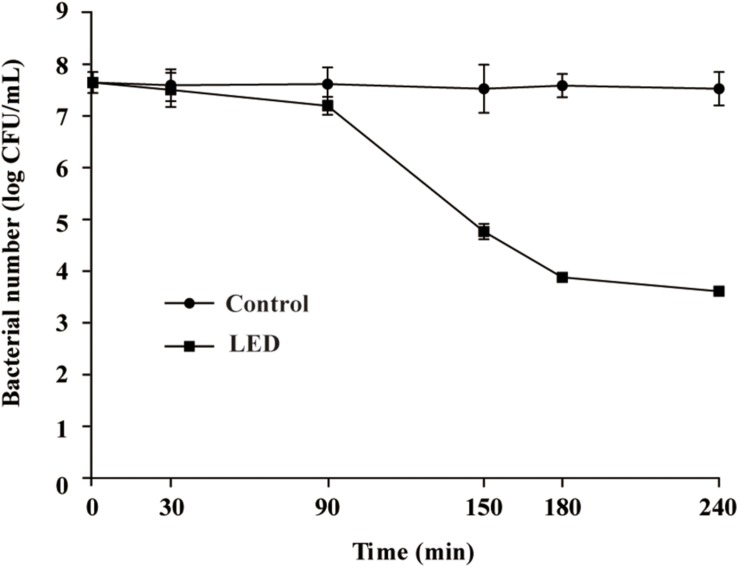
Effect of 405-nm LED illumination on the survival of *L. monocytogenes* suspensions at 4°C.

Changes in the survival of *L. monocytogenes* illuminated with the 405-nm LED are shown in Figure 1. The initial concentration of *L. monocytogenes* was 7.8 log CFU/mL. Following illumination at 4°C for 90, 150, or 180 min, the concentration of *L. monocytogenes* cells was reduced by 0.5, 2.9, and 3.6 log CFU/mL, respectively, whereas the populations in the unilluminated samples fluctuated around 7.8 log CFU/mL. The bacterial numbers in the LED-illuminated and unilluminated samples differed significantly (*P* < 0.01) after LED illumination for 150 min. Therefore, illumination for 150 min (a total dose of 170 J/cm^2^) was chosen as the sample treatment to study the effects of LED illumination on the resistance of *L. monocytogenes* to different environmental stresses.

### Environmental Stress Resistance of *L. monocytogene*s

#### Resistance of *L. monocytogenes* to Oxidative Stress

**FIGURE 2:**
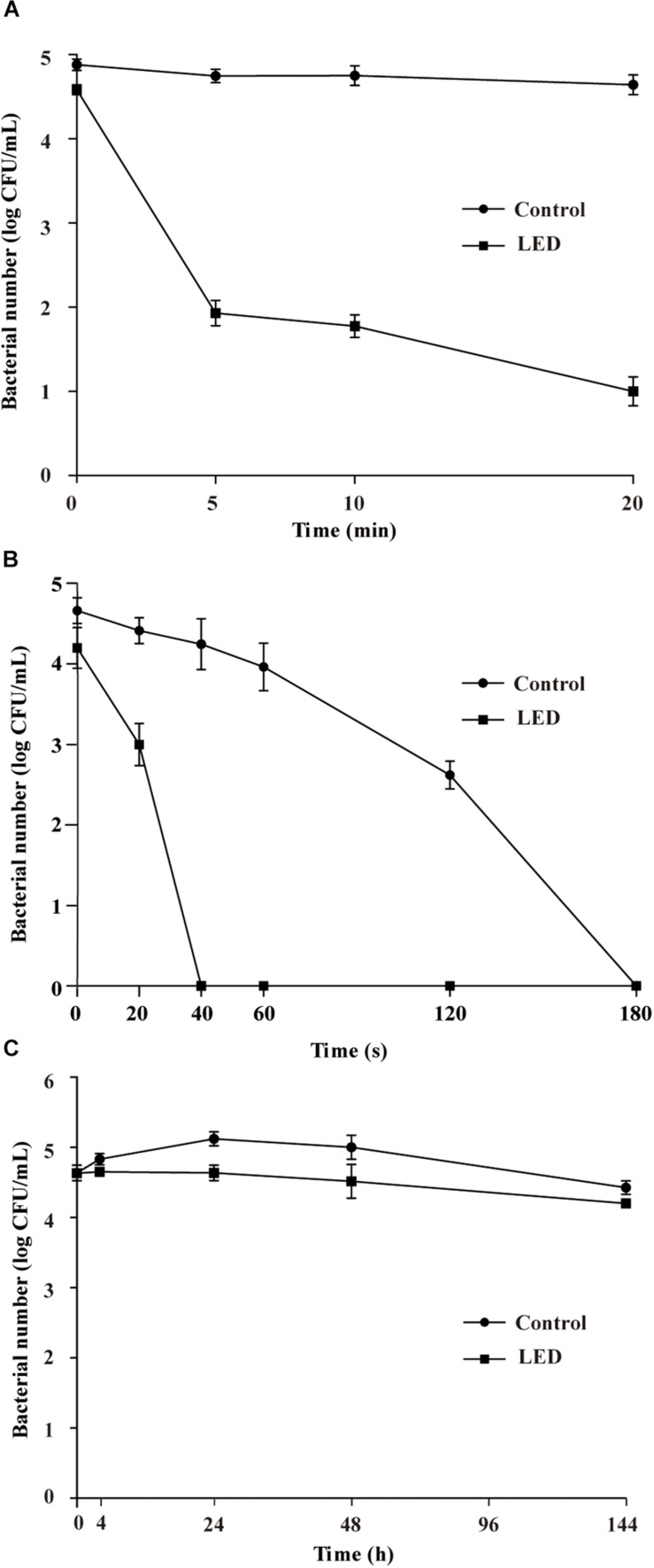
Survival of LED-illuminated and unilluminated *L. monocytogenes* cultures in 0.04% H_2_O_2_ **(A)**, under UV irradiation **(B)**, and at 4°C **(C)**.

The effects of 405-nm LED treatment on the oxidative stress resistance of *L. monocytogenes* are shown in Figure 2A. Bacterial concentrations in the unilluminated samples remained at about 4.8 log CFU/mL after a 20-min exposure to 0.04% H_2_O_2_. In contrast, *L. monocytogenes* concentrations in the illuminated samples decreased rapidly in the first 5 min, resulting in a significant differences (*P* < 0.01) in concentration compared with the unilluminated samples. After this time point, bacterial concentrations remained constant (1.8 log CFU/mL) until the end of the experimental period.

#### UV Resistance of *L. monocytogene*s

Figure 2B shows the effect of 405-nm LED treatment on the resistance of *L. monocytogenes* to UV irradiation. The bacterial population decreased significantly (*P* < 0.01) after UV irradiation. Following UV irradiation for 40, 60, or 120 s, the numbers of bacteria in the unilluminated samples decreased by about 0.4, 0.7, and 2.0 log CFU/mL, respectively, and decreased to below the detection limit after 180 s. Decreases in bacterial numbers in the illuminated cultures following 20 s of UV irradiation were significantly (*P* < 0.01) greater than those observed in the unilluminated cultures, with population numbers dropping below the detection limit within 40 s.

#### Cold Resistance of *L. monocytogene*s

The initial concentrations of illuminated and unilluminated bacteria were about 4.6 log CFU/mL (Figure 2C). In the unilluminated samples refrigerated at 4°C for 24 h, the *L. monocytogenes* counts increased by about 0.5 log CFU/mL, but decreased to 4.4 log CFU/mL after 96 h. When illuminated with LED, the *L. monocytogenes* counts decreased slightly in the first 24 h, falling to 4.2 log CFU/mL after 96 h. There was a significant difference (*P* < 0.01) between the illuminated and unilluminated cultures in the first 24 h, but no significant difference was noted between the culture types after 24 h (*P* > 0.05).

#### Resistance of *L. monocytogenes* to Osmotic Pressure

**FIGURE 3:**
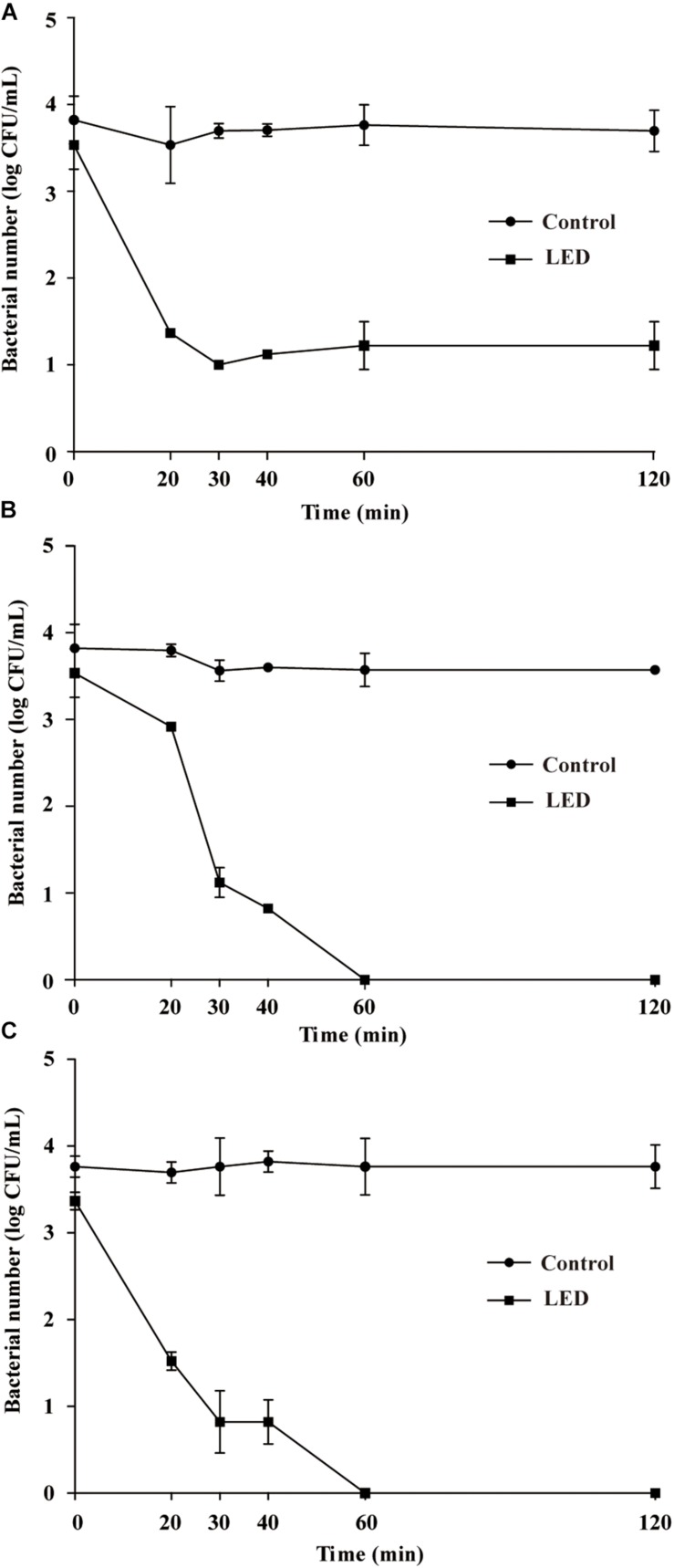
Survival of LED-illuminated and unilluminated *L. monocytogenes* in 10% (w/v) NaCl **(A)**, 15% (w/v) NaCl **(B)**, and 20% (w/v) NaCl **(C)**.

The survival of LED-illuminated *L. monocytogenes* cultures exposed to 10, 15, and 20% (w/v) NaCl is shown in Figures 3A–C, respectively. After the NaCl solutions were inoculated with the samples, the concentration of *L. monocytogenes* was 3.6 log CFU/mL in each sample. *L. monocytogenes* concentrations in the unilluminated samples remained at around 3.6 log CFU/mL regardless of the NaCl concentration. In comparison, illuminated bacterial sample concentrations significantly decreased following exposure to 10% NaCl (*P* < 0.01), decreasing to 2.3 log CFU/mL after 20 min and 1.1 log CFU/mL after 20–120-min exposure times (Figure 3A). The concentrations of LED-illuminated *L. monocytogenes* cultures incubated in 15 and 20% NaCl for 30 min decreased to about 1.1 and 0.9 log CFU/mL, respectively, and dropped below the detection limit after 60 min (Figures 3B,C).

#### Resistance of *L. monocytogenes* to SGF

**FIGURE 4:**
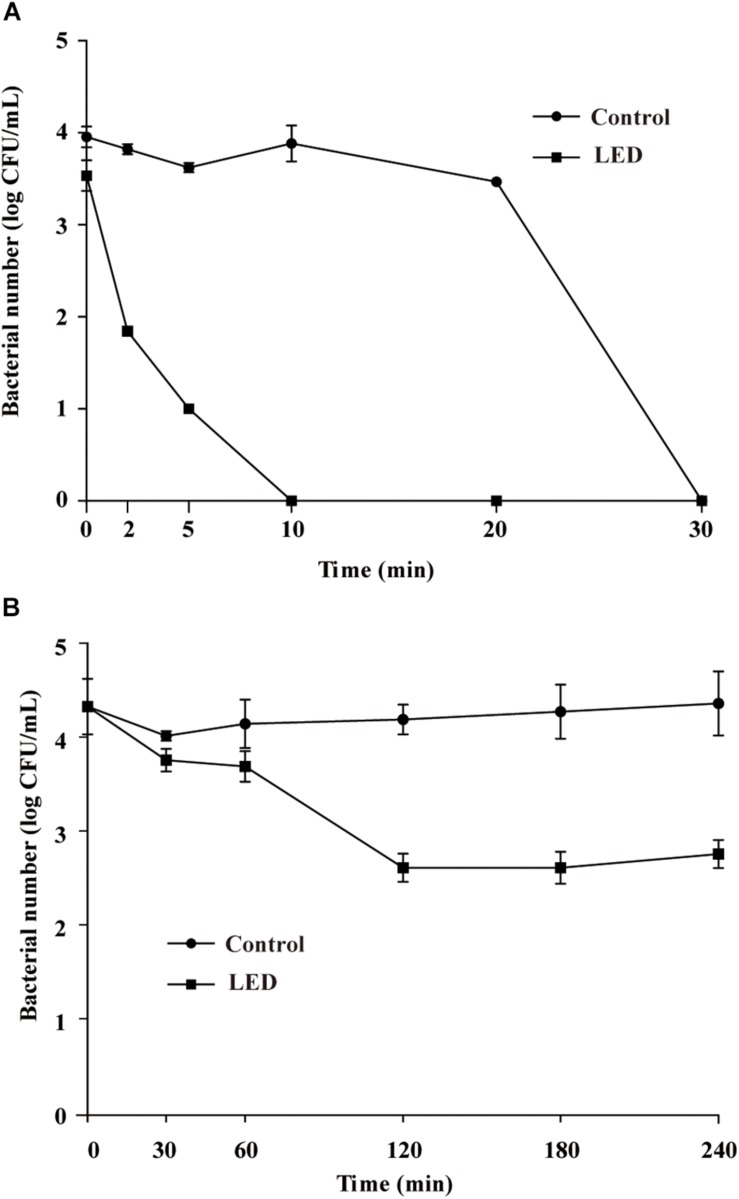
Survival of LED-illuminated and unilluminated *L. monocytogenes* in SGF at pH 2.5 **(A)**, and in 2% (w/v) bile salt solution **(B)**.

Figure 4A shows the effects of 405-nm LED treatment on the resistance of *L. monocytogenes* to SGF. After exposure to SGF for 5, 10, or 20 min, the numbers of bacteria in the unilluminated populations decreased by about 0.3, 0.1, and 0.5 log CFU/mL, respectively, and dropped below the detection limit after 30 min. Cell concentrations in the LED-illuminated *L. monocytogenes* samples were significantly reduced (*P* < 0.01) compared with the unilluminated samples in response to SGF, and dropped below the detection limit after a 10 min-exposure period.

#### Resistance of *L. monocytogenes* to Bile Salts

Figure 4B shows the effects of LED treatment on the bile-salt resistance of *L. monocytogenes*. The cell concentrations in the unilluminated samples remained at 4.2 log CFU/mL throughout the experimental period. However, cell counts in the illuminated *L. monocytogenes* samples decreased by approximately 1.7 log CFU/mL after 120 min in bile salts, but remained unchanged from 120–240 min.

### Effects of 405-nm LED Treatment on the Integrity of *L. monocytogenes* Cell Membranes

**FIGURE 5:**
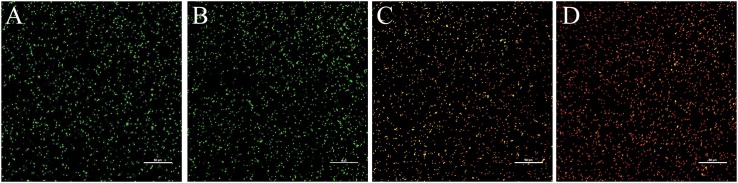
Confocal laser scanning microscope images of *L. monocytogenes*. Untreated bacterial cells incubated for 90 min **(A)** or 150 min **(B)**, and bacterial cells treated with LED illumination for 90 min **(C)** or 150 min **(D)** were visualized under the confocal laser scanning microscope.

The effects of 405-nm LED treatment on cell membrane integrity in *L. monocytogenes* is shown in Figure 5. Unilluminated *L. monocytogenes* cells showed green fluorescence at 90 and 150 min post-inoculation, indicating intact cells. However, bacteria treated with LED illumination for 90 min displayed yellow and red fluorescence, indicating that the cell membrane had been disrupted. *L. monocytogenes* cells illuminated with the 405-nm LED for 150 min showed almost no green fluorescence, with an abundance of red fluorescence in the field of view. This result indicated that 405-nm LED treatment impacted the cell membrane integrity of *L. monocytogenes*, and that the degree of damage increased with the period of illumination.

### Effects of 405-nm LED Treatment on Gene Transcription in *L. monocytogene*s

**FIGURE 6:**
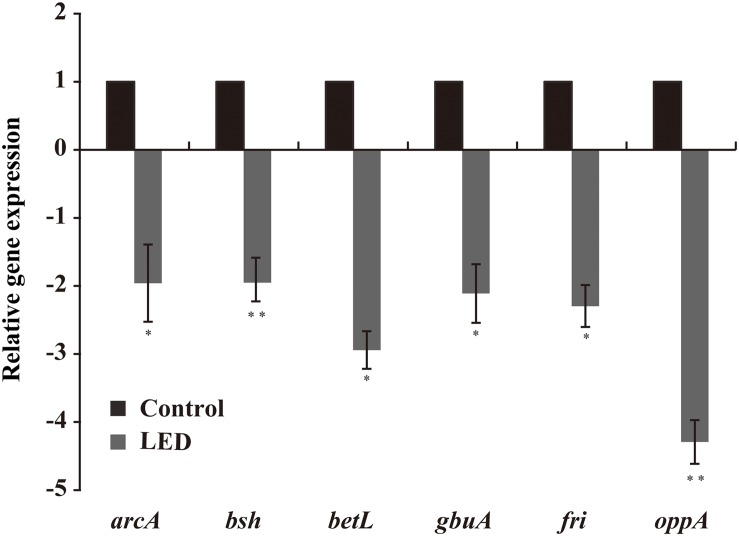
Effects of LED treatment on the transcription of genes associated with the resistance of *L. monocytogenes* to environmental stresses. *^∗^P* < 0.05 and *^∗∗^P* < 0.01 versus the control.

As shown in Figure 6, LED treatment had a significant effect on the transcription of environmental-stress-related genes in *L. monocytogenes*. Transcription of *betL* and *gbuA*, which are associated with osmotic stress, was downregulated following LED illumination, as was that of *oppA* and *fri* (critical for cold resistance), to various degrees. Other downregulated genes included *bsh* (critical for bile-salt resistance) and *arcA* (critical for acid resistance).

## Discussion

*Listeria monocytogenes* persists under a wide range of stress conditions (NicAogáin and O’Byrne, 2016). Food-related stress conditions encountered by *L. monocytogenes* along the food chain arise from various methods of preservation, and include oxidative stress generated by disinfectants, UV stress during sterilization, cold stress caused by refrigeration, and osmotic stress caused by increased salt concentrations (Bucur et al., 2018). Then, when *L. monocytogenes* enters the host’s gastrointestinal tract, it encounters the acidic environment of the stomach and bile salts in the intestine, before ultimately invading host cells and triggering serious disease (Gahan and Hill, 2014). Therefore, reducing the resistance of *L. monocytogenes* to stress conditions is crucial for preventing contamination and controlling its growth along the entire food chain, from production to consumption. A previous study demonstrated that *L. monocytogenes* was reduced by 5 log CFU/mL when irradiated with 108 J/cm^2^ of illumination using a 405-nm LED (Murdoch et al., 2012), while we confirmed that the concentration of *L. monocytogenes* cells was reduced by 2.9 log CFU/mL following exposure to 170 J/cm^2^ of illumination using a 405-nm LED. In the present study, we investigated the ability of 405-nm LED treatment to reduce the resistance of *L. monocytogenes* to food-related stress conditions.

Chemical oxidizing reagents, such as detergents and disinfectants, are often used to clean raw food materials and industrial equipment to prevent and control foodborne pathogens (Abeysundara et al., 2016). *L. monocytogenes* can survive for more than 15 min after exposure to the oxidative agent cumene hydroperoxide at a concentration of 13.8 mM (Ferreira et al., 2001). In the present study, 0.04% H_2_O_2_ was used to simulate an oxidative environment, with results showing that the resistance of *L. monocytogenes* to oxidative stress was significantly reduced by 405-nm LED illumination. Los and Murata (1997) reported that the resistance of *L. monocytogenes* to 0.1% H_2_O_2_ was significantly enhanced after preincubation at acidic pH levels or pretreatment with weak organic acids. After exploring the mechanisms involved, the researchers found that the expression of sigma factor σ^*S*^ (KatF) was increased and that synthesis of catalases HPI (encoded by *katG*) and HPII (encoded by *katE*) was induced. In the current study, we observed that the transcription of *fri* was reduced by LED illumination. *fri* contributes to *L. monocytogenes* survival in oxidative and low-iron environments because it encodes ferritin, which detoxifies oxidative agents (Olsen et al., 2005). Therefore, the reduction in the oxidative resistance of *L. monocytogenes* induced by 405-nm LED treatment may be attributable to its effect on the abundance of ferritin.

Ultraviolet irradiation is another method used to control *L. monocytogenes* and other foodborne pathogens during food decontamination (Gómez-López et al., 2007). However, *L. monocytogenes* can persist under UV irradiation and is more resistant than *Salmonella* or *E. coli* O157:H7 when suspended in PBS or clear apple juice (Gabriel and Nakano, 2009). This difference in resistance may be related to differences in the DNA repair mechanisms used by these bacteria to combat DNA damage caused by UV light, such as photoreactivation by the enzyme photolyase and nucleotide excision repair (Sinha and Häder, 2002). We demonstrated that the number of LED-illuminated bacteria dropped below the limit of detection after UV illumination for 20 s, confirming that 405-nm LED treatment significantly reduces the survival of *L. monocytogenes* following UV irradiation. A similar study showed that UV radiation is more lethal to *Salmonella* when combined with heat treatment, and suggested that this is related to a reduction in cell repair or to additional lethal damage caused by the interaction of the by-products induced by the two agents (Gayán et al., 2012). However, Gayán et al. (2015) recently reported that previous exposure to sublethal doses of heat (48°C, 1 h, pH 7.0), acidity (pH 4.5, 1 h), or alkalinity (pH 9.0, 1 h) did not significantly affect the resistance of *L. monocytogenes* to UV.

Preservation under refrigeration is a common method of food storage. However, *L. monocytogenes* can proliferate to an effective concentration at 4°C (Tasara and Stephan, 2006). In the present study, LED illumination effectively reduced the cold resistance of *L. monocytogenes* to less than 24 h. Marcos et al. (2008) reported that the cold resistance of *L. monocytogenes* can be reduced by high-pressure processing (400 MPa, 10 min), and showed that treated bacteria do not regenerate to viable cells during long-term refrigeration. The cold resistance of *L. monocytogenes* is related to the oligopeptide permease (*opp*) operon (Borezee et al., 2000). The *oppA* gene product plays a major role in the uptake of oligopeptides and is necessary for the growth of the bacterium at low temperatures (Cabrita et al., 2015). *oppA* transcription was downregulated by 405-nm LED treatment in the present study, indicating that LED treatment reduces the cold resistance of *L. monocytogenes* by affecting the expression of the *opp* operon.

The ability of *L. monocytogenes* to survive or proliferate under osmotic pressure is key to its survival and growth during food production (Bucur et al., 2018). Al-Nabulsi et al. (2015) demonstrated that *L. monocytogenes* can persist in highly osmotic environments (10–20% (w/v) NaCl) because it accumulates osmolytes to reduce the osmotic pressure caused by water loss. Our results showed that 405-nm LED treatment reduced the resistance of *L. monocytogenes* to the osmotic pressure exerted by 10, 15, and 20% (w/v) NaCl solutions. A previous study reported that compared with control samples, the bacterial counts of trans-cinnamaldehyde-pretreated *Cronobacter sakazakii* cultures decreased by more than 100,000-fold after treatment with 75% (w/v) sorbitol for 36 h. The researchers attributed this to the downregulation of the outer membrane proteins (OmpC, OmpR, and OsmY) involved in the transport of osmoprotectants across the bacterial cytoplasmic membrane (Amalaradjou and Venkitanarayanan, 2011). Wemekamp-Kamphuis et al. (2002) reported that the growth and survival of *L. monocytogenes* at high osmotic pressures are mainly attributable to the accumulation of glycine betaine and carnitine with *gbu* and *betL* encoding the main glycine betaine transporters of *L. monocytogenes*. We demonstrated that LED treatment significantly reduced the transcription of *gbuA* and *betL* potentially explaining why *L. monocytogenes* illuminated using 405-nm LED showed lower resistant to osmotic stress than unilluminated cells.

Prior to invading host cells, *L. monocytogenes* is faced with the hostile environment of the gastrointestinal tract, which is characterized by a low pH and is recognized as the first line of defense against foodborne pathogens (Cotter et al., 2001). Various mechanisms allow *L. monocytogenes* to survive and even replicate under acidic conditions, including glutamic acid decarboxylase, F0F1-ATPase, and arginine (Bucur et al., 2018). In this study, the exposure of *L. monocytogenes* to LED illumination reduced its survival in SGF (pH 2.5). Similarly, the survival of trans-cinnamaldehyde-pretreated *C. sakazakii* cells was reduced in acidified TSB or infant formula milk (pH 3.3), with a >10-fold difference in bacterial counts following exposure to trans-cinnamaldehyde for 60 min (Amalaradjou and Venkitanarayanan, 2011). Li et al. (2013) showed that heat shock at 47°C for 15 min enhanced the acid resistance of *C. sakazakii* by inducing the expression of stress-related proteins. The acid-stress-related gene *arcA* was downregulated by 405-nm LED illumination in the present study. ArcA activates and promotes the arginine deiminase pathway, which imports arginine molecules from the extracellular environment and protonates them to ammonium (NH_4_) to increase the cellular pH (Cotter and Hill, 2003; Cheng et al., 2013). Therefore, the reduced acid resistance of *L. monocytogenes* may be attributable to the effect of 405-nm LED treatment on the arginine deiminase pathway.

Bile in the intestines disorganizes the structure of the bacterial membrane and triggers DNA damage, making it harder for *L. monocytogenes* to survive in and colonize the gastrointestinal tract (Hsiao et al., 2010). However, *L. monocytogenes* can replicate in the lumen of the gall bladder, where bile salt concentrations can reach up to 15% (Dowd et al., 2011). The human intestine contains a bile salt concentration gradient ranging between 2 and 0.05% under normal physiological conditions (Ruiz et al., 2013). Therefore, we tested the effect of LED illumination on the resistance of *L. monocytogenes* to a 2% bile salt solution. The number of viable LED-illuminated cells decreased faster than that of unilluminated cells at a bile salt concentration of 2% under normal physiological conditions. Similarly, Wright et al. (2016) showed that a moderate adjustment of the oxygen concentration reduced the bile-salt resistance of *L. monocytogenes* by affecting the proteomes of strains F2365, EGD-e, 10403S, and HCC23. However, Hsiao et al. (2010) reported that heat shock at 47°C for 15 min did not affect the resistance of *C. sakazakii* to a bile salt solution. *bsh* encodes bile brine hydrolase, and its deletion reduces the resistance of *L. monocytogenes* to bile salts (Dussurget et al., 2010). In current study, we showed that the transcription of *bsh* was downregulated by LED illumination. Therefore, we postulate that LED treatment reduces the bile-salt resistance of *L. monocytogenes* by affecting the abundance of bile brine hydrolase.

Many foodborne pathogens can survive under unfavorable conditions because they are protected by cell membranes (Beales, 2004). The main mechanism by which LED treatment inactivates microbes involves its stimulation of bacteria to produce ROS, which damage both cell membranes and proteins (Maclean et al., 2009). Therefore, we investigated changes in the integrity of the bacterial cell membrane following LED illumination. We observed that 405-nm LED illumination disrupted cell membrane integrity and reduced the resistance of *L. monocytogenes* to environmental stresses. Similar to our results, Kim et al. (2019) reported that cell membrane damage was significantly greater in *E. coli* O157:H7 at a low growth temperature, observing decreases in the resistance of the pathogen to ohmic heating as the growth temperature decreased from 37 to 15°C. The cell membrane enhances the survival of bacteria under adverse conditions by altering its fluidity (Beales, 2004). Proteins in the cell membrane also play significant roles in the resistance of bacteria to environmental stresses. For example, outer membrane proteins (OmpC, OmpR, and OsmY) are involved in the transport of osmotic protectants in the cell membrane of *Enterobacter sakazakii* (Riedel and Lehner, 2010). Sudden changes in environmental conditions such as heat shock, osmotic stress, and freezing can cause changes in the cell membrane structure, including membrane damage (Los and Murata, 2004). Therefore, we assume that the destruction of the cell membrane integrity influences the resistance of *L. monocytogenes* to environmental stresses.

## Conclusion

The results of the present study demonstrate that 405-nm LED illumination reduces the resistance of *L. monocytogenes* to several environmental stresses. The numbers of LED-illuminated bacteria decreased significantly compared with the control when exposed to oxidative stress (0.04% H_2_O_2_), UV irradiation (253.7 nm), low temperature (4°C), osmotic pressure (10, 15, or 20% NaCl), SGF (pH 2.5), and bile salts (2%). CLSM and RT-qPCR analyses showed that LED illumination reduced the integrity of the bacterial cell membrane and downregulated the expression of several genes associated with environmental stress resistance. Our findings shed new light on the use of LED illumination as a promising, safe treatment for effectively controlling *L. monocytogenes* in the food production industry. However, the ability of 405-nm LED treatment to reduce the resistance of other *L. monocytogenes* strains to environmental stresses, along with the effects of the treatment on the *in vivo* and *in vitro* pathogenicity of *L. monocytogenes* must be further explored to evaluate the practical effects and feasibility of using 405-nm LED to control *L. monocytogenes* and reduce the risk of human infection.

## Data Availability

The raw data supporting the conclusions of this manuscript will be made available by the authors, without undue reservation, to any qualified researcher.

## Author Contributions

SK, YM, and CS conceived and designed the experiments. DG, XC, and JT performed the experiments. SK and YM analyzed the data. YX, SL, and XX contributed to reagents, materials, and analysis tools. SK, YM, XC, and JT wrote the manuscript.

## Conflict of Interest Statement

The authors declare that the research was conducted in the absence of any commercial or financial relationships that could be construed as a potential conflict of interest.

## Supplementary Material

FIGURE S1 Temperature profile of *L. monocytogenes* suspension during 405-nm LED illumination within acrylonitrile butadiene styrene housing at 4°C at a distance of 4.5 cm from the light source.
