# Supplementary figures and images for: Effects of 405-nm LED Treatment on the Resistance of Listeria monocytogenes to Subsequent Environmental Stresses
Source: Front Microbiol. 2019 Aug 16;10:1907. doi: 10.3389/fmicb.2019.01907 (PMC6706791; doi:10.3389/fmicb.2019.01907)

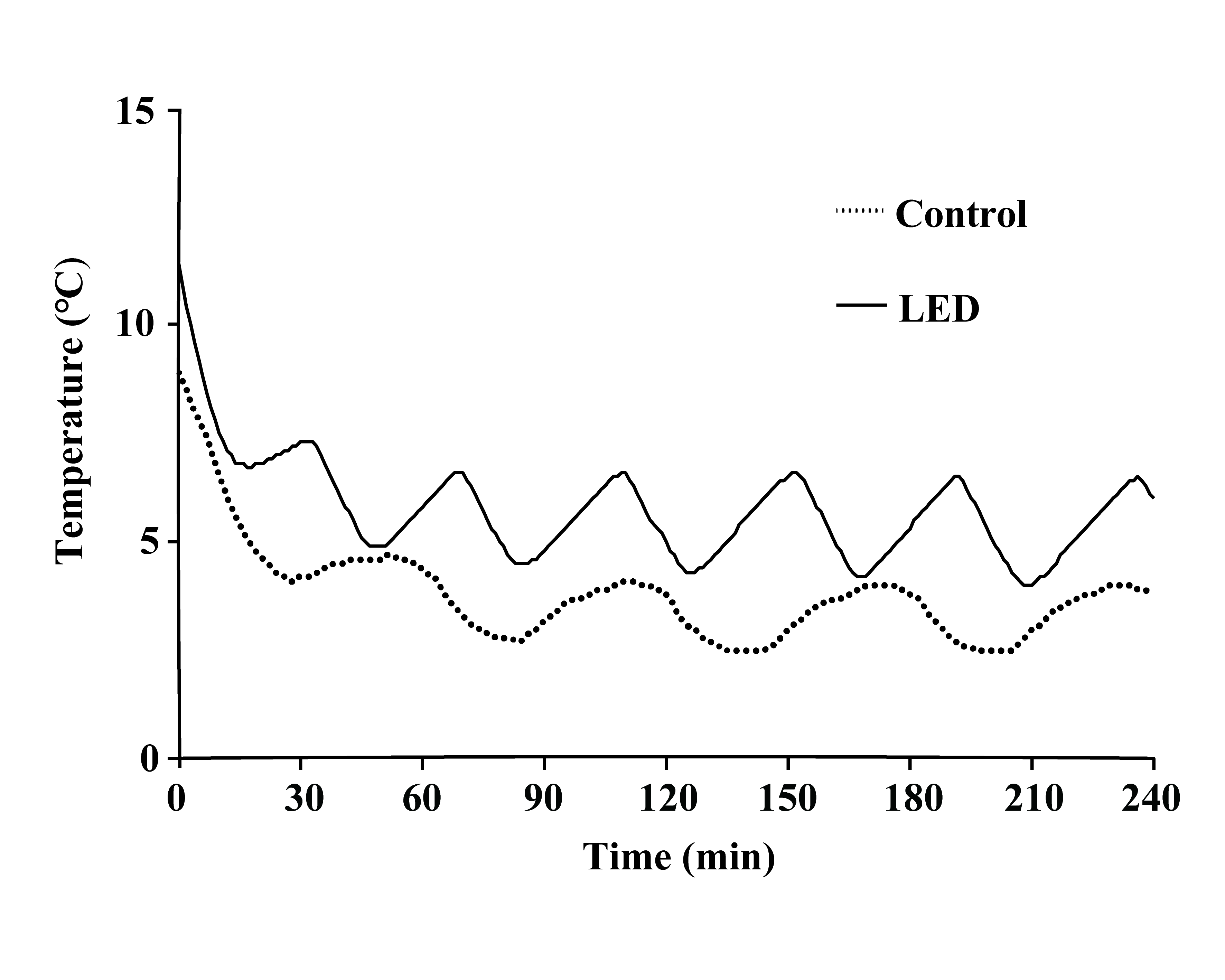

Supplement: FIGURE S1 — Temperature profile of L. monocytogenes suspension during 405-nm LED illumination within acrylonitrile butadiene styrene housing at 4°C at a distance of 4.5 cm from the light source. [file Image_1.TIF]
